# Highly Sensitive HBsAg, Anti-HBc and Anti HBsAg Titres in Early Diagnosis of HBV Reactivation in Anti-HBc-Positive Onco-Haematological Patients

**DOI:** 10.3390/biomedicines10020443

**Published:** 2022-02-14

**Authors:** Carlotta Cerva, Romina Salpini, Mohammad Alkhatib, Vincenzo Malagnino, Lorenzo Piermatteo, Arianna Battisti, Ada Bertoli, Jeff Gersch, Vera Holzmayer, Mary Kuhns, Gavin Cloherty, Ludovica Ferrari, Campogiani Laura, Elisabetta Teti, Maria Cantonetti, William Arcese, Francesca Ceccherini-Silberstein, Carlo-Federico Perno, Massimo Andreoni, Valentina Svicher, Loredana Sarmati

**Affiliations:** 1 I.N.M.I. “Lazzaro Spallanzani”, 00149 Rome, Italy; carlottacerva@gmail.com; 2 Department of Experimental Medicine, Tor Vergata University, 00133 Rome, Italy; r.salpini@gmail.com (R.S.); mohammad--alkhatib@hotmail.com (M.A.); piermatteolorenzo@gmail.com (L.P.); battisti.arianna@gmail.com (A.B.); bertoli@uniroma2.it (A.B.); ceccherini@med.uniroma2.it (F.C.-S.); valentina.svicher@uniroma2.it (V.S.); 3 Unit of Clinical Infectious Disease, Department of System Medicine, Tor Vergata University, 00133 Rome, Italy; malagninovincenzo@gmail.com (V.M.); ludovicaferrari@hotmail.it (L.F.); lauracampg@gmail.com (C.L.); elisabetta.teti@gmail.com (E.T.); andreoni@uniroma2.it (M.A.); 4 Infectious Disease Research, Abbott Diagnostics, Abbott Park, Green Oaks, IL 60064, USA; jeffrey.gersch@abbott.com (J.G.); Vera.Holzmayer@abbott.com (V.H.); mary.kuhns@abbott.com (M.K.); Gavin.Cloherty@abbott.com (G.C.); 5 Stem Cell Transplant Unit, Department of Hematology, Tor Vergata University, 00133 Rome, Italy; cantonetti@med.uniroma2.it (M.C.); william.arcese@ptvonline.it (W.A.); 6 Children Hospital “Bambino Gesù”, 00165 Rome, Italy; cf.perno@uniroma2.it

**Keywords:** HBV reactivation, anti-HBc positivity, resolved HBV infection, oncohaematological patients, new HBV markers, antiviral prophylaxis, HSCT

## Abstract

The role of novel HBV markers in predicting Hepatitis B virus reactivation (HBV-R) in HBsAg-negative/anti-HBc-positive oncohaematological patients was examined. One hundred and seven HBsAg-negative/anti-HBc-positive oncohaematological patients, receiving anti-HBV prophylaxis for >18 months, were included. At baseline, all patients had undetectable HBV DNA, and 67.3% were anti-HBs positive. HBV-R occurred in 17 (15.9%) patients: 6 during and 11 after the prophylaxis period. At HBV-R, the median (IQR) HBV-DNA was 44 (27–40509) IU/mL, and the alanine aminotransferase upper limit of normal (ULN) was 44% (median (IQR): 81 (49–541) U/L). An anti-HBc > 3 cut-off index (COI) plus anti-HBs persistently/declining to <50 mIU/mL was predictive for HBV-R (OR (95% CI): 9.1 (2.7–30.2); 63% of patients with vs. 15% without this combination experienced HBV-R (*p* < 0.001)). The detection of highly sensitive (HS) HBsAg and/or HBV-DNA confirmed at >2 time points, also predicts HBV-R (OR (95% CI): 13.8 (3.6–52.6); 50% of positive vs. 7% of negative patients to these markers experienced HBV-R (*p* = 0.001)). HS-HBs and anti-HBc titration proved to be useful early markers of HBV-R. The use of these markers demonstrated that HBV-R frequently occurs in oncohaematological patients with signs of resolved HBV infection, raising issues of proper HBV-R monitoring.

## 1. Introduction

HBsAg-negative/anti-HBc-positive patients with oncohaematological diseases are at high risk of hepatitis B virus reactivation (HBV-R). This is due to the intrahepatic persistence of HBV circular covalently closed DNA (cccDNA) coupled with profound, and often durable, immunosuppression [1,2,3,4].

HBV-R has a negative impact on the clinical course of oncohaematological patients, since it is associated with liver injury and with delayed administration or premature termination of immunosuppressive treatments, contributing to worsening prognosis [1]. For this reason, most international guidelines recommend starting antiviral prophylaxis in oncohaematological patients at high HBV-R risk. Nevertheless, defining the most appropriate drug and duration for antiviral prophylaxis remains challenging, with important differences in recommendations across international guidelines [2,3,4,5]. However, HBV-R cases, occurring even several years after the last immunosuppressive therapy, have been frequently reported, particularly in patients undergoing haematological stem cell transplantation (HSCT), as a consequence of deep and long-lasting immunosuppression [6]. This highlights the need to better define proper antiviral prophylaxis in this special population.

Recently, novel biomarkers have been proposed to reflect the intrahepatic HBV reservoir [7,8], providing information on the presence of active cccDNA. In this context, a promising biomarker is represented by anti-HBc titres. Notably, in immunocompetent anti-HBc-positive/HBsAg-negative individuals, anti-HBc titres with a >4 cut-off index (COI) has been associated with the presence of intrahepatic cccDNA [9], while in HIV-positive patients, anti-HBc titres with a >15 COI is suggestive of cryptic HBV replication [10].

Moreover, serum HBV-RNA emerged as a novel marker reflecting cccDNA transcriptional activity [11]. This biomarker, measuring the number of virions containing pregenomic HBV-RNA (thus lacking the reverse transcriptase process) [12], is particularly useful in unravelling ongoing viral activity in patients on antiviral prophylaxis and/or treatment.

The availability of these innovative markers also parallels the development of novel assays with increased sensitivity for detecting/quantifying HBV biomarkers (such as serum HBsAg and HBV-DNA) capable of identifying minimal HBV replicative activity, thus allowing an early HBV-R diagnosis and reducing the risk of developing severe clinical manifestations [13,14].

In this light, this study aims to (i) evaluate the rate of HBV-R in HBsAg-negative/anti-HBc-positive oncohaematological patients undergoing antiviral prophylaxis for >18 months; and (ii) define the role of highly sensitive detection of HBsAg (HS-HBs) and quantitative anti-HBc titres in predicting HBV-R in the setting of haematological malignancies. In a subgroup of patients treated for HBV-R, the trend in the HBV-RNA values was also evaluated.

## 2. Materials and Methods

### 2.1. Study Population

This longitudinal study included 107 adult patients followed for a haematological malignancy at the University Hospital Tor Vergata, Rome, Italy, from January 2016 to December 2020. Patients were HBsAg negative/anti-HBc positive and HBV-DNA negative at baseline screening. Patients received lamivudine prophylaxis for >18 months after completing chemotherapy and/or HSCT according to EASL Guidelines [15]. Patients were monitored every 3 months to assess HBV-R occurrence during or after completing HBV prophylaxis for a median (IQR) duration of 44 (31–56) months. All data were censored in December 2020, with a target monitoring period of 2 years. HBV-R was defined as HBV-DNA reappearance (HBV-DNA ≥ 20 IU/mL), regardless of liver biochemistry [16].

All the patients’ personal information was treated in a confidential manner and all clinical data were collected anonymously to respect the patients’ privacy. Informed consent to study participation was obtained from all enrolled subjects. All methods were performed in accordance with the relevant guidelines and regulations. The study was approved by the Tor Vergata University Ethical Committee (Code: RS.178.18).

### 2.2. HBV Laboratory Evaluation

#### 2.2.1. Classical HBV Markers

HBsAg was tested by the commercially available quantitative HBsAg detection system (Abbott Architect, lower limit of detection (LLoD): 50 mIU/mL (routinely used for diagnostic purposes at University Hospital Tor Vergata) (Abbott Diagnostics, Abbott Park, IL, USA). Similarly, the qualitative detection of anti-HBc and anti-HBs titres was assessed by the Abbott Architect instrument (Abbott Diagnostics, Abbott Park, IL, USA). Serum HBV DNA was quantified by a real-time PCR system (COBAS Amplicor-COBAS TaqMan, Roche, Basel, Switzerland; lower limit of quantitation (LLoQ): 20 IU/m).

#### 2.2.2. Innovative HBV Markers

##### HS-HBs

HS-HBs was assessed by an ARCHITECT HBsAg NEXT Qualitative assay (HBsAgNx; Abbott Diagnostics, Abbott Park, IL, USA, LLoD: 5 mIU/mL), while the highly sensitive quantification of HBsAg was by Lumipulse HBsAg-HQ (Fujirebio) (LLOQ: 5 mIU/mL), working on a LUMIPULSE^®^G system for the qualitative assay (Abbott).

Both assays are characterized by a 1 log IU/mL increase in sensitivity with respect to serological assays routinely used for HBsAg detection/quantification. HBsAg detection by both assays showed a high concordance (98.1%).

##### Quantification of Anti-HBc Titer

Anti-HBc levels were quantified by using the Lumipulse^®^G HBcAb-N assay (Fujirebio, Tokyo, Japan). The assay is based on a fully automated two-step sandwich CLEIA technology working on the LUMIPULSE^®^G system. Anti-HBc IgG levels are automatically reported as COI, calculated as a multiple of the cut-off value obtained from the calibration data (COI = S/C × 0.09). The lower limit of quantification reported by the manufacturer for the assay was 1 COI.

##### Quantification of Serum HBV-RNA

The levels of serum pregenomic HBV-RNA were assessed by using the Abbott Real-Time HBV-RNA Research Use Only assay (Abbott Diagnostics, Abbott Park, IL, USA), as described in [17]. Results are expressed as log10 U/mL. The lower limit of quantitation of the assay was 1.65 log10 U/mL. Serum HBV-RNA was quantified in 51 samples from 6 HBV-reactivated patients at the diagnosis of HBV-R and during the following antiviral therapy (median (IQR) time of treatment: 12 (11–24) months; median (IQR) number of samples collected for patient: 3 (2–6)).

### 2.3. Population-Based Sequencing of HBV Reverse Transcriptase

Population-based sequencing of HBV reverse transcriptase was performed on serum samples at HBV-R [18].

In details, HBV-DNA was extracted using a commercially available kit (QIAmp DNAblood mini-kit, Qiagen Inc., Germantown, MD, USA), and amplified with Amplitaq-Gold-polymerase using the following primer pairs: 5′-GGTCACCATATTCTTGGGAA and 5′-GTGGGGGTTGCGTCAGCAAA. PCR conditions were: one cycle (93 °C 12 min), 40 cycles (94 °C 50 s, 57 °C 50 s, 72 °C 1 min and 30 s). PCR-products were sequenced by using eight different sequence-specific primers, BigDye-terminator-v.3.1 sequencing kit (Applied-Biosystems Foster City, CA, USA ) and an automated sequencer (ABI-3130 XL). The sequences were analysed by using SeqScape software. The quality endpoint was ensured by a double sequence coverage for each nucleotidic region.

### 2.4. Statistical Methods

The Mann–Whitney U-test for continuous variables and Fisher’s exact test for discrete variables were applied to define statistically significant differences. The cumulative probability of experiencing HBV-R was estimated by Kaplan–Meier analysis in the overall population and in patients stratified according to immunosuppressive intervention. Independent factors associated with HBV-R were assessed by logistic regression analysis. Only the factors with a *p* value < 0.200 by univariate analysis were included in the multivariate model. Statistical analyses were performed by IBM SPSS software.

## 3. Results

### 3.1. Study Population

This study included 107 patients with a diagnosis of an oncohaematological disease. Most patients were male (57%) and Italian (88.8%), with a median (IQR) age of 66 (58–77) years (Table 1). The most frequent haematological malignancy was non-Hodgkin lymphoma (NHL) (36.5%), followed by acute myeloid leukaemia (AML) (15.9%), multiple myeloma (MM) (14.0%), and chronic lymphocytic leukaemia (CLL) (12.1%) (Table 1). Most patients received rituximab-containing chemotherapy (39.2%), while 40 (37%) patients underwent HSCT as part of the therapy course. Among them, 32 (80%) underwent allogeneic HSCT.

At HBV baseline screening, before starting the immunosuppressive regimen, patients were HBsAg negative and anti-HBc positive. Most of them (79.3%) were also positive for anti-HBs (median (IQR) titre: 152 (47–976) mIU/mL) (Table 1). At the end of the study evaluation, 61/107 (57%) patients completed the recommended course of anti-HBV prophylaxis (Table S1).

### 3.2. Occurrence of HBV-R

**Figure 1:**
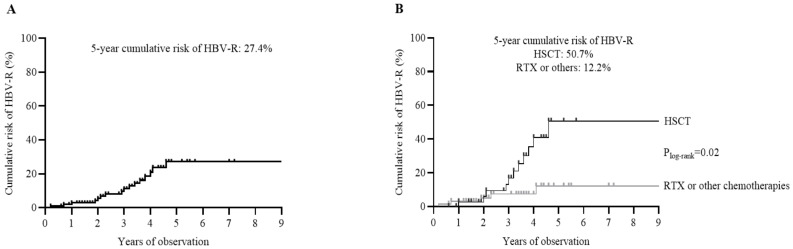
Survival analysis by competing risk estimates of the cumulative odds of HBV reactivation. The graph plots report the cumulative risk for the occurrence of HBV-reactivation in overall population (N = 107) (**A**) and in patients stratified according to HSCT (N = 40) versus RTX (N = 42) and/or other chemotherapies (N = 25) (**B**). The cumulative risk was calculated by Kaplan Meier. Statistically significant difference was calculated by log-rank test.

During the entire patients’ monitoring (median (IQR duration) time: 44 [31–56] months), HBV-R was observed in 17/107 (15.8%) patients, with a 5-year cumulative risk of 27.4% (Table 2 and Figure 1A). The 5-year cumulative risk of HBV-R was significantly higher in patients undergoing HSCT (without statistically significant differences between autologous and allogenic HSCT: 54.3% vs. 51.1%) than in those receiving chemotherapies with or without rituximab (Figure 1B).

Notably, most cases of HBV-R (11/17, 64.7%) occurred after completing anti-HBV prophylaxis (range: 1–27 months after prophylaxis completion). In the remaining 35.3%, HBV-R occurred while receiving antiviral prophylaxis (Table 2). No patients harboured drug-resistant strains.

At HBV-R, the median (IQR) HBV-DNA was 44 (27–40,509) IU/mL, and ALT was above the normal level (>UNL) in 44% of patients (median (IQR) ALT: 81 (49–541) U/L), indicating a diagnosis of biochemical HBV-R in less than half of the patients (Table 2). These data are indicative of an early HBV-R diagnosis as a result of strict patient monitoring (every 3 months) followed in this study protocol. The median anti-HBc titre was 22 (3–46) COI.

The graph plots report the cumulative risk for the occurrence of HBV reactivation in the overall population (N = 107) (A) and in patients stratified according to HSCT (N = 40) versus chemotherapies with (N = 42) or without rituximab (RTX) (N = 25) (B). The cumulative risk was calculated by Kaplan–Meier analysis. Statistically significant differences were calculated by the log-rank test.

### 3.3. Predictive Role of Serological HBV Markers in the Diagnosis of HBV-R

**Figure 2:**
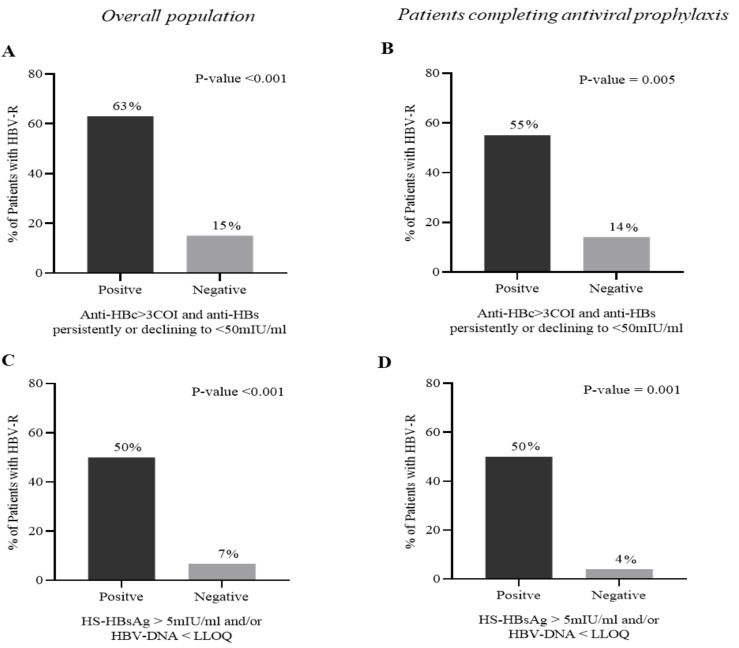
The combined usage of accurate HBV markers can predict the occurrence of HBV reactivation. The histogram reports the occurrence of HBV-reactivation in patients positive/negative to anti-HBc > 3COI + anti-HBs < 50 mIU/mL (**A**,**C**) and in patients positive/negative HS-HBsAg and/or HBV-DNA (**B**,**D**). Data in (**A**,**B**) are referred to overall population (N = 107), while in (**C**,**D**) are referred to the subset of patient completing the recommended course of antiviral prophylaxis (N = 61) during the entire follow-up. Statistically significant difference was calculated Chi-squired test for independence based on 2 × 2 contingency table.

An anti-HBc > 3 COI combined with an anti-HBs persistent or declining to <50 mIU/mL during patient monitoring was significantly correlated with a higher risk of developing HBV-R (OR (95% CI): 9.1 (2.7–30.2); *p* < 0.001). Indeed, 63% of patients with the combination of an anti-HBc > 3 COI plus anti-HBs < 50 mIU/mL experienced HBV-R compared to 15% of patients without this combination (*p* < 0.001) (Figure 2A). Notably, this result was also confirmed in the subset of patients experiencing HBV-R after completing anti-HBV prophylaxis (OR (95% CI): 8.8 (2.0–38); *p* = 0.005). In this setting, 55% of patients with the combination of an anti-HBc > 3 COI plus anti-HBs < 50 mIU/mL experienced HBV-R versus 14% without this combination (*p* = 0.005) (Figure 2B).

A further step in this study was to evaluate the predictive role of virological markers in HBV-R diagnosis. The on-monitoring analysis revealed that the detection of HS_HBs and/or of serum HBV-DNA (target detected below LLOQ), confirmed at least at 2 time points, was a predictive factor for HBV-R (OR (95% CI): 13.8 (3.6–52.6); *p* < 0.001). Indeed, 50% of patients positive to HS-HBsAg and/or to serum HBV-DNA versus 7% never positive to these markers experienced HBV-R (*p* < 0.001) (Figure 2C). This result was also confirmed in the subset of patients experiencing HBV-R after completing anti-HBV prophylaxis (*p* < 0.001) (Figure 2D), suggesting that monitoring these biomarkers could be useful to identify patients requiring prolonged or enhanced prophylaxis.

Multivariate analysis confirmed that either the combination of anti-HBc > 3 COI+ anti-HBs < 50 mIU/mL or positivity for HS-HBsAg and/or serum HBV-DNA (target detected below LLOQ) were independent predictive factors for HBV-R (OR (95% CI): 7.2 (1.4–39.2), *p* = 0.020, and 5.3 (1.0–27.8), *p* = 0.049, respectively) (Table 3).

The histogram reports the occurrence of HBV reactivation in patients with or without the combination of anti-HBc > 3 COI + anti-HBs < 50 mIU/mL (A,B) and in patients positive/negative for HS-HBsAg and/or HBV DNA (C,D). Data in (A,C) refer to the overall population (N = 107), while those in (B,D) refer to the subset of patients completing the recommended course of antiviral prophylaxis (N = 61) during the entire follow-up. Statistically significant differences were calculated using the chi-square test for independence based on a 2 × 2 contingency table.

### 3.4. Outcome of Patients Experiencing HBV-R

Among the 17 patients experiencing HBV-R, 16 received antiviral therapy with entecavir and/or tenofovir disoproxil fumarate/tenofovir alafenamide and were monitored for a median (IQR) time of 19 (15–34) months, while one patient was lost to follow-up.

By analysing the treatment outcome, ALT normalization was achieved by all patients after a median (IQR) time of 3 (3–9) months, and 81.3% (13/16) achieved virological suppression within 6 months of antiviral therapy.

Overall, 25% of patients (4/16) died from the progression of the underlying oncohaematological disease, while no death due to hepatic failure occurred.

In a subset of 6 (35%) out of 17 patients, serum HBV RNA was also quantified at HBV-R and during antiviral treatment (median (IQR) time of follow-up: 12 (11–24) months). At HBV-R, serum HBV-RNA ranged from <LLOQ to 6.6 log IU/mL. In 5 out of 6 patients, both serum HBV-DNA and HBV-RNA became undetectable within 6 months of treatment and remained undetectable in the subsequent follow-up, suggesting rapid and durable silencing of cccDNA transcriptional activity. A different scenario was observed in the remaining patient, characterized at HBV-R by serum HBV-DNA and HBV-RNA of 6.0 log IU/mL and 6.6 log IU/mL, respectively. Notably, after 9 months of treatment, a significant decay (up to 2.8 log IU/mL) was observed exclusively for serum HBV-DNA, while serum HBV-RNA remained stable at approximately 6 log IU/mL, indicating elevated cccDNA transcriptional activity. To date, the patient is still in entecavir treatment, and the last (June 2021) HBV-DNA value was undetectable below 20 UI/mL. He is continuing regular follow up.

### 3.5. The Added Value of HS HBsAg Quantification: A Case Report

Here, a clinical case is reported, highlighting the importance of HS-HBsAg quantification in unravelling minimal HBV replicative activity.

**Figure 3:**
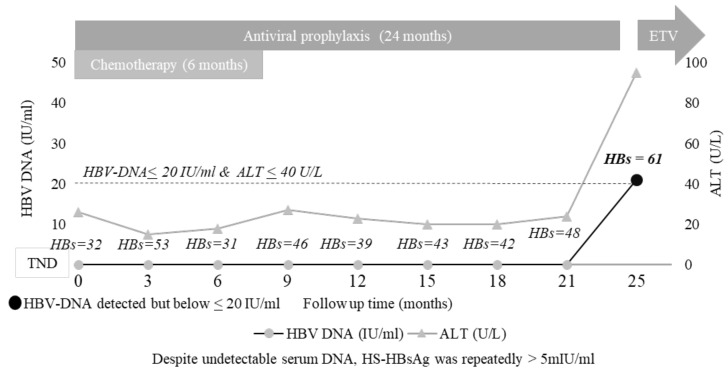
A clinical case shows the quantified persistence of high-sensitivity HBsAg.

The patient was a 58-year-old Italian male diagnosed with Hodgkin lymphoma. The patient was anti-HBc, isolated at baseline screening, and thus received proper antiviral prophylaxis before starting chemotherapy, containing adriamycin, bleomycin, vinblastyn, and dacabarzin. During 24 months of patient monitoring, serum HBV-DNA was persistently undetectable, and HBsAg was persistently negative by classical assays, while transaminases fell within the normal values. Antiviral prophylaxis was suspended 18 months after completing chemotherapy. Unexpectedly, 1 month after prophylactic suspension, the patient experienced HBV-R with HBV-DNA and HBsAg reappearance in serum and ALT levels rising to 95 IU/mL (Figure S1). The highly sensitive quantification of HBsAg revealed that HBsAg was already detected at each time point analysed during antiviral prophylaxis, supporting the intrahepatic activity of the HBV reservoir that can explain HBV-R occurrence immediately after the suspension of antiviral prophylaxis (Figure 3).

The line chart reports the trend of serum HBV-DNA and ALT during patient monitoring. At each time point, the highly sensitive detection of HBsAg was assessed by an ARCHITECT HBsAg NEXT Qualitative assay (HBsAgNx; Abbott Diagnostics, Abbott Park, IL, USA, LLoD: 5 mIU/mL), while the highly sensitive quantification of HBsAg by Lumipulse HBsAg-HQ (Fujirebio) (LLOQ: 5 mIU/mL), working on a LUMIPULSE^®^G system the qualitative assay (Abbott).

## 4. Discussion

This study shows the occurrence of HBV-R in almost 16% of oncohaematological anti-HBc-positive/HBsAg-negative patients. Notably, most of them (64.7%) experienced HBV-R after completing the recommended course of antiviral prophylaxis, supporting the need to reconsider its proper duration in a setting characterized by profound immunosuppression. These results may pave the way for designing further ad hoc studies aimed to define a new desirable duration of antiviral prophylaxis.

At the same time, this study raises the issue to unravel the role of novel HBV biomarkers in identifying patients at higher risk of HBV-R. In this direction, this study shows that the combination of an anti-HBc > 3 COI plus anti-HBs at or declining to <50 mIU/mL during the follow-up is predictive for HBV-R. This datum, confirmed by multivariable analysis, is also present in the subgroup of patients experiencing HBV-R after prophylaxis interruption. Our finding is in keeping with a previous study on an Asian cohort of oncohaematological patients showing that the levels of anti-HBc > 6.41 IU/mL and anti-HBs < 56.48 mIU/mL may predict HBV-R in patients with lymphoma [19].

Overall, these findings support the concept that the progressive weakening of the humoral response coupled with transcriptionally active cccDNA can play an important role in driving HBV-R. This corroborates the importance of integrating HBV biomarkers, reflecting the activity of the intrahepatic HBV reservoir and the strength of anti-HBV immune responses, for the optimized management of severely immunosuppressed patients at risk of HBV-R. In particular, the combination of an anti-HBc > 3 COI plus anti-HBs < 50 mIU/mL could guide the identification of patients more prone to develop HBV-R who need closer monitoring, extended prophylaxis, or more potent anti-HBV drugs, such as tenofovir or entecavir.

Furthermore, the on-monitoring analysis of virological markers showed that the detection of HS-HBs and/or a target detected serum HBV-DNA (below LLOQ) strongly increases HBV-R risk. This result, confirmed by multivariate analysis, is present in patients who reactivated HBV both during prophylaxis and after its suspension, thus demonstrating the ability of these biomarkers to identify patients with minimal viral replication. The role of HS-HBs is supported by the paradigmatic clinical case showing persistent positivity for this biomarker (not detected by the currently available assays) preceding HBV-R. Such positivity was also coupled with persistently high anti-HBc levels (ranging from 64 to 130 COI), further reinforcing the presence of an already replicative active HBV reservoir, even several months before the clinical evidence of HBV-R diagnosis. This finding is also consistent with a recent Japanese study showing that the detection of HS-HBs and a target detected serum HBV-DNA below the lower limit of quantification can help identify patients more prone to HBV-R [20].

In this context, highly sensitive assays for HBV-DNA quantification, such as those based on digital droplet PCR, capable of detecting even a few copies of HBV-DNA [14,21], could also represent a useful tool to demonstrate the presence of low-level HBV replicative activity.

Although based on a limited sample size, this study also supports the utility of serum HBV-RNA (although not yet available in clinical practice) in monitoring the therapeutic response of patients experiencing HBV-R. The loss of serum HBV-RNA, observed in most patients during treatment, may indicate exhausting or transcriptional silencing of cccDNA. As reported by previous studies, this highlights the potential role of serum HBV-RNA as a biomarker to guide the safe discontinuation of NUC therapy [22].

In the present study, almost half of patients with HBV-R underwent allogeneic HSCT, which represents a category of patients characterized by the highest grade of severe immunosuppression. Little information is available on the history of immune recovery in our study population, but it is known that it may take years post-HSCT. In this light, assessing this issue could help to better define the correlation between the degree and quality of immunosuppression and HBV-R risk.

In conclusion, HBV-R frequently occurs in anti-HBc-positive/HBsAg-negative oncohaematological patients, particularly after completing the recommended course of antiviral prophylaxis, suggesting the need to reconsider the duration of prophylaxis in profound immunosuppression. In this setting, close monitoring based on the integrated use of novel HBV markers can help to detect minimal viral replication, thus guiding the identification of patients at higher risk of developing HBV-R who would need to change the anti-HBV drug towards more potent antivirals (tenofovir, entecavir) or to extend prophylaxis.

## Figures and Tables

**Table 1:** Characteristics of the study population.

Patients’ Characteristics	N = 107
Male/Female, N (%)	61/46 (57/43)
Age (years), median (IQR)	66 (58–77)
Italian origin, N (%) Duration of monitoring, median (IQR) months	95 (88.8)
	44 (31–56)
Oncohaematological Diseases, N (%)	
Non-Hodgkin Lymphoma (NHL)	39 (36.5)
Acute Myeloid Leukaemia (AML)	17 (15.9)
Multiple Myeloma (MM)	15 (14.0)
Chronic Lymphocytic Leukaemia (CLL)	13 (12.1)
Hodgkin Lymphoma (HL)	6 (5.6)
Acute Lymphocytic Leukaemia (ALL)	5 (4.7)
Other Diseases ^a^	12 (11.2)
Immunosuppressive Regimens, N (%)	
Chemotherapy + Rituximab	42 (39.2)
Allogenic HSCT	32 (29.9)
Autologous HSCT	8 (7.5)
Other Chemotherapies	25 (23.4)
HBV Serological Profiles	
Anti-HBc positive anti-HBs negative, N (%)	22 (20.7%)
Anti-HBc positive anti-HBs positive, N (%)	85 (79.3%)
Anti-HBs titre, median (IQR) mIU/mL	152 (47–976)
Antiviral Prophylaxis	
Use of antiviral prophylaxis, N (%)	107 (100)
Patient still in antiviral prophylaxis	39 (36)
Patient who interrupted antiviral prophylaxis	61 (57)

^a^ Amyloidosis, Sézary syndrome, autoimmune haemolytic anaemia, T-lymphoblastic leukaemia/lymphoma, myelodysplastic syndrome, chronic myelogenous leukaemia, systemic mastocytosis, and mycosis fungoides. Abbreviation: HSCT, hematopoietic stem cell transplantation; anti-HBs, antibodies against hepatitis B surface antigen; anti-HBc, antibodies against hepatitis B core antigen.

**Table 2:** Characteristics of 17 patients at HBV-R.

Patients’ Characteristics	N = 17
Serum HBV-DNA, median (IQR) IU/mL	44 (27–40,509)
Serum ALT > UNL, N (%)	7 (44)
- Serum ALT, median (IQR) U/L	81 (49–541)
HBV Serological Profiles at HBV-R	
HBsAg positive, N (%):	4 (23.5)
HBsAg negative, N (%)	13 (76.5)
Anti-HBs positive, N (%):	8 (47)
- Anti-HBs titre, range mIU/mL	13–505
Immunosuppressive Regimen, N (%)	
Chemotherapy + Rituximab 3 (17.6)	
Allogeneic HSCT 8 (47.2)	
Autologous HSCT 3 (17.6)	
Other Chemotherapies 3 (17.6)	
HBV reactivation occurrence	
- During prophylaxis, N (%)	6 (35.3)
- After prophylaxis completion, N (%)	11 (64.7)
Months after prophylaxis completion, median (IQR)	4 (2–13)

Abbreviations: ALT, alanine aminotransferase; UNL; upper normal limit; HBsAg; hepatitis B surface antigen; anti-HBs, antibodies against hepatitis B surface antigen; HSCT, hematopoietic stem cell transplantation.

**Table 3:** Factors associated with HBV-R by multivariable logistic regression analysis.

Variables ^a^	Univariable Analysis		Multivariable Analysis	
	Crude OR (95% CI)	*p*-Value	Adjusted OR (95% CI)	*p*-Value
Gender (male vs. female)	2.0 (0.7–6.2)	0.224	-	-
Age (for 1 year increase)	1.0 (1.0–1.1)	0.101	1.1 (1.0–1.1)	0.435
HSCT vs. RTX and other chemotherapies	3.9 (1.3–11.5)	**0.015**	3.0 (0.5–17.2)	**0.018**
Combination of anti-HBc > 3 COI + anti-HBs < 50 mIU/mL	9.1 (2.7–30.2)	**<0.001**	7.2 (1.4–39.2)	**0.020**
Detection to HS-HBs and/or to serum HBV-DNA < 20 IU/mL	13.8 (3.6–52.6)	**<0.001**	5.3 (1.0–27.8)	**0.049**

^a^ The logistic regression analysis was performed on 107 oncohaematological anti-HBc-positive/HBsAg-negative patients. The following variables were considered: gender, age, HSCT vs. RXT and/or other chemotherapies, combined anti-HBc > 3 COI + anti-HBs < 50 mIU/mL, and positivity to HS-HBs or HBV-DNA detected <20 IU/mL. Only variables showing a *p*-value ≤ 0.200 in the univariable analysis were included in the multivariable analysis. Abbreviations: HSCT, hematopoietic stem cell transplantation; RTX, Rituximab; OR, odds ratio; CI, confidence interval; anti-HBs, antibodies against hepatitis B surface antigen; anti-HBc, antibodies against hepatitis B core antigen; HS-HBs, high-sensitive hepatitis B surface antigen. The significant values appeared in bold.

## Data Availability

Data is contained within the article and supplementary material.

## Supplementary Materials

The following supporting information can be downloaded at: https://www.mdpi.com/article/10.3390/biomedicines10020443/s1, Table S1: Characteristics of patients completing anti HBV prophylaxis.
