# Supplementary material for: Highly Sensitive HBsAg, Anti-HBc and Anti HBsAg Titres in Early Diagnosis of HBV Reactivation in Anti-HBc-Positive Onco-Haematological Patients
Source: Biomedicines. 2022 Feb 14;10(2):443. doi: 10.3390/biomedicines10020443 (PMC8962433; doi:10.3390/biomedicines10020443)
Supplement: Supplementary file 1 [file biomedicines-10-00443-s001.zip › biomedicines-1575751-supplementary.pdf]

Table S1. Characteristics of patients completing anti HBV prophylaxis.

Patients'characteristics	*N=61*
Male, N (%)	33 (54.1)
Age [Years], Median (IQR)	66 (57-73)
Italian Origin, N (%)	54 (88.5)
Oncohaematological Diseases, N (%)	
Non-Hodgkin Lymphoma (NHL)	22 (36.1)
Acute Myeloid Leukemia (AML)	13 (21.3)
Multiple Myeloma (MM)	8 (13.1)
Chronic Lymphocytic Leukemia (CLL)	6 (9.8)
Hodgkin Lymphoma (HL)	5 (8.2)
Acute Lymphocytic Leukemia (ALL)	3 (4.9)
Other Diseases[a]	4 (6.6)
Immunosuppressive Regimens, N (%)	
Rituximab	23 (37.7)
Allogenic HSCT	20 (32.8)
Autologous HSCT	6 (9.8)
Other Chemotherapies	12 (19.7)
HBV serological profiles	
Anti-HBc positive anti-HBs negative, N (%)	20 (32.8%)
Anti-HBc positive anti-HBs positive, N (%)	41 (67.2%)
Anti-HBs titer, Median (IQR) mIU/ml	165 (47-969)
Antiviral Prophylaxis	
Duration of Antiviral Prophylaxis, Median (IQR) Months	25 (19-39)

[a] Sézary syndrome, Autoimmune haemolytic anaemia, and Mycosis fungoides.

Abbreviation: HSCT, hematopoietic stem cell transplantation; anti-HBs, antibodies against hepatitis B surface antigen; anti-HBc, antibodies against hepatitis B core antigen.
